# Physical activity and psychosocial characteristics of individuals with and without chronic low back pain in daily life: protocol for the PRIA intensive longitudinal study

**DOI:** 10.1136/bmjopen-2025-109887

**Published:** 2025-11-29

**Authors:** Karolina Kolodziejczak-Krupp, Valerie Zipper, Lea O Wilhelm, Lara Thiel, Christoph Stein, Thomas Schäfer, Matthias Pumberger, Hendrik Schmidt, Lena Fleig

**Affiliations:** 1 Department of Psychology, MSB Medical School Berlin GmbH, Berlin, Germany; 2 Department of Education and Psychology, Freie Universität Berlin, Berlin, Germany; 3 Department of Anaesthesiology and Intensive Care Medicine, Charité - Universitätsmedizin Berlin, Campus Benjamin Franklin, Berlin, Germany; 4 Department of Psychology, HMU Health and Medical University Erfurt GmbH, Erfurt, Germany; 5 Center for Musculoskeletal Surgery, Charité - Universitätsmedizin Berlin, Berlin, Germany; 6 Julius Wolff Institute, Berlin Institute of Health, Berlin, Germany

**Keywords:** Back pain, PUBLIC HEALTH, Primary Prevention

## Abstract

**Introduction:**

Despite the high prevalence of chronic low back pain (cLBP), its underlying mechanisms remain poorly understood. Addressing modifiable psychosocial resources and health behaviours such as physical activity offers a promising avenue for reducing the impact of cLBP. Furthermore, although the relationship between physical activity and pain is theorised as a within-person process, previous research has primarily focused on between-person differences. In this article, we present the protocol for the prospective observational study PRIA (Psychologie und Rückengesundheit im Alltag), which is part of a larger interdisciplinary research consortium investigating preventive, diagnostic and therapeutic aspects of cLBP. Drawing on theories from health and pain psychology, the outlined study examines the interplay between different dimensions of cLBP and back health, physical activity and their psychosocial determinants within individuals in their everyday lives.

**Methods and analysis:**

This prospective longitudinal study combines online questionnaires with ecological momentary assessment of health behaviours, cognitions, affect, social support and pain using a smartphone-based app (movisensXS) and continuous measurement of physical activity by accelerometry (movisens Move 4). Parameters will be recorded at baseline (T0), daily for the following 14 days (five times per day at 09:00, 12:00, 15:00, 18:00 and 21:00, resulting in up to 70 measurement occasions), 3 and 6 months later (T1 and T2). A total of 230 participants (115 individuals with cLBP and 115 without cLBP) aged 18–64 years will be enrolled. The associations between cLBP and the measured parameters will be examined using multilevel models.

**Ethics and dissemination:**

The university’s ethics committee at the MSB Medical School Berlin approved the study on 8 March 2021 (approval number MSB-2021/59, amendment approved on 10 November 2023, amendment number MSB-2023/145). Ethical approval for the FOR 5177 initial screening was granted by Charité – Universitätsmedizin Berlin (EA1/058/21). All participants provided written informed consent. The results of this research will be published in peer-reviewed international journals, presented at national and international conferences, and reported to the German Research Foundation.

**Trial registration number:**

DRKS00032978.

**STRENGTHS AND LIMITATIONS OF THIS STUDY**

- This study shifts the focus from a pathological perspective to a more resource-oriented approach to chronic low back pain (cLBP), with the aim of preventing cLBP and promoting back health.
- The study considers psychosocial risk factors and resources alongside biomechanical factors in a cohort of individuals with and without cLBP.
- Ecological momentary assessment combined with accelerometry allows for high temporal data resolution at the intra-individual level.
- The observational design of the study precludes conclusions about causality; instead, the study highlights key risk factors and resources for cLBP that should be examined further in interventional studies, for example, in the form of just-in-time adaptive interventions.

## Introduction

 Low back pain (LBP) is a health complaint responsible for the most years lived with disability worldwide.^1^ A population-based study from Germany showed that 52.9% of adults had experienced LBP in the past year, with 15.5% of this cohort reporting back pain for at least 3 months, referred to as chronic back pain.^2^ Despite the high prevalence of chronic LBP (cLBP) and its substantial contribution to work-related disability,^3^ understanding of the aetiology of cLBP and its successful prevention remains limited. Recent research suggests that targeting modifiable behaviour-related risk factors such as smoking, high body mass index (BMI) and low physical activity could substantially reduce the number of years lived with disability due to cLBP.^1 4^ This emphasises the importance of prioritising health behaviours such as physical activity in the prevention and treatment of cLBP. Therefore, this study aims to examine patterns of physical activity and their linkages with cLBP. Additionally, we aim to identify psychosocial predictors of pain, back health and physical activity, grounded in behaviour change theory, to inform the development of behavioural interventions.

### The role of psychosocial factors and physical activity in cLBP

According to the biopsychosocial model of LBP,^5^ cLBP is considered a multidimensional syndrome. Often, the persistence of pain is not directly related to the factors that contributed to the initial onset of pain, such as injury or overuse, but rather to the factors that maintain the pain. As non-specific LBP (ie, back pain without a clear organic origin) persists, psychological, behavioural and social risk factors may contribute to its progression to cLBP.^6 7^ Similarly, a growing body of literature shows that the subjective experience of pain (eg, pain intensity) is highly dependent on psychological risk factors—commonly referred to as ‘yellow flags’. These include, for example, perceived stress, negative affect, depressive symptoms and cognitive responses to pain such as catastrophising.^7 8^

In turn, engaging in health behaviour such as physical activity is essential for enhancing overall health and lowering the risk of mortality.^9^ The WHO recommends undertaking regular physical activity, including moderate-to-vigorous intensity physical activity and muscle-strengthening physical activity in a considerable amount throughout the week.^10^ Although physical activity, and leisure-time physical activity (LTPA) in particular,^11 12^ is widely acknowledged as an important non-pharmacological strategy for preventing and managing cLBP, there is inconsistent and limited evidence regarding the most beneficial type, intensity, duration and frequency of LTPA for preventing cLBP.^11 13^

### Understanding the links between pain, psychosocial factors and physical activity: towards a resource-oriented approach to cLBP

While previous research has often focused on risk factors, such as health risk behaviours^1^ or maladaptive cognitions,^8^ the present study takes a more comprehensive approach that includes both risk and protective factors.^14^ Such an approach emphasises that the absence of disease cannot be equated with health and posits that LBP and back health are distinct concepts.^15^

**Figure 1:**
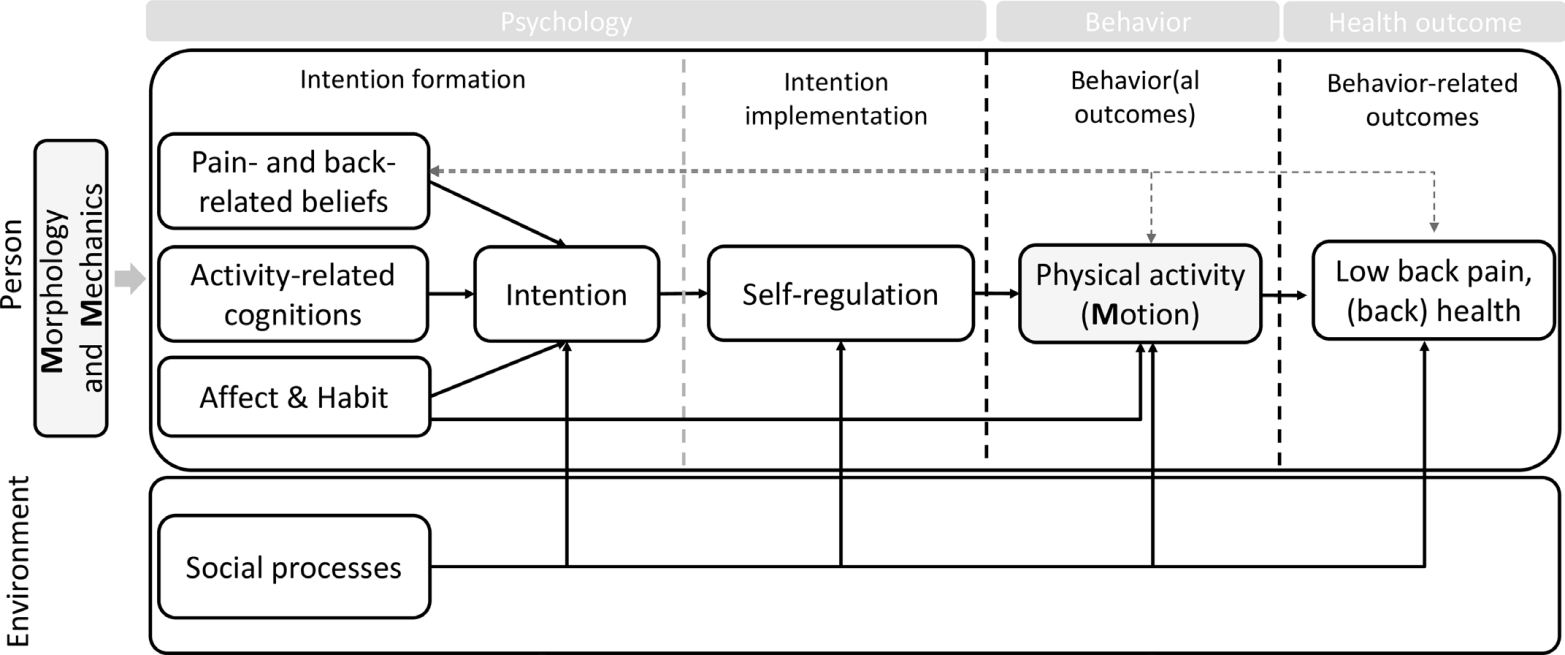
Theoretical framework of the PRIA study: The Back Health Behaviour Model by Wilhelm *et al.*^16^ The figure has been adapted to include biomechanical variables: spine morphology, mechanics and motion (‘The 3 M’s’). PRIA, Psychologie und Rückengesundheit im Alltag.

Theoretical perspectives from the psychology of behaviour change and the chronification of pain provide a framework for examining the complex relationships between health or disease, psychological and social factors, and health behaviours. In particular, the back health behaviour model (BHBM)^16^ represents a systematic, theory-based framework that integrates key insights from pain psychology (ie, the fear-avoidance model^17^ and the avoidance-endurance model of pain^18^) and health psychology (ie, the health action process approach, HAPA^19^ and the physical activity adoption and Maintenance^20^). As the BHBM considers both psychological and behavioural resources and risk factors (see figure 1), it is well suited to a comprehensive examination of cLBP and back health.

Taking a closer look at the BHBM,^16^ at the behavioural level, health behaviour such as LTPA can be seen as a resource-oriented coping and prevention strategy. Importantly, successful engagement in health behaviours requires the involvement of explicit and implicit processes at the psychological level. For instance, an individual’s intention to engage in physical activity is determined by their subjective appraisal of their own resources (eg, (pain) self-efficacy, (pain-related) social support), but also by their emotional states (eg, affect) or pain-related beliefs (eg, fear-avoidance beliefs). In turn, if individuals are motivated to adopt behavioural changes, they need to translate their intentions into behaviour using volitional, self-regulatory strategies (eg, planning). Figure 1 shows the BHBM modified to account for contextual and biomechanical factors.

### Capturing fluctuations in pain intensity, back health and physical activity using ecological momentary assessment

Both the experience of pain and related factors often vary over time within individuals.^21^ Thus, multiple assessment designs that aim to capture the temporal dynamics between variables are well suited to examining cLBP. The number of studies investigating cLBP using ecological momentary assessment (EMA) designs has increased over time.^22^ An EMA design can provide new insights into how psychosocial predictors, health behaviours (ie, physical activity), and behaviour-related outcomes (ie, pain intensity, back health) evolve at different levels of temporal resolution and with different time lags, for example, moment-to-moment or day-to-day. This, in turn, allows us to test theoretical assumptions at both the within-person and between-person levels simultaneously.^23^

### The present study

Studies examining the associations between physical activity and pain and its theory-based, modifiable predictors are needed to inform the design of behavioural interventions to prevent cLBP. This prospective observational study PRIA (Psychologie und Rückengesundheit im Alltag) considers both risk factors and resources for cLBP, both in terms of health behaviours (eg, LTPA, back posture and mobility) and psychosocial factors (eg, self-efficacy, positive affect, social support). Importantly, in line with health conceptualisations,^15^ self-reports of LBP are complemented by self-reports of back health to explore the potential utility of health measures in the prevention and treatment of cLBP. Another important aim of the PRIA study is to investigate the intra-individual (ie, within-person) variation in pain, back health and physical activity and its theoretical determinants. In doing so, the study aims to improve our understanding of how the temporal dynamics between LBP and back health evolve in the daily lives of individuals with and without cLBP, and how these within-person patterns differ between individuals with different individual characteristics.

Specifically, the research objectives of the PRIA study are to (a) capture how LBP intensity and back health unfold in daily life, (b) identify physical activity patterns in individuals with and without cLBP, (c) examine the associations between pain intensity, back health and physical activity and (d) identify key modifiable psychosocial correlates of and health behaviours related to pain intensity and back health. While seeking modifiable correlates of cLBP, we will also consider the role of non-modifiable factors. For example, given the accumulating evidence that sex and gender differences are an important source of variation in how individuals experience and respond to pain,^24 25^ we will consider gender to build a comprehensive model.

#### Between-person hypotheses

As physical activity, and LTPA in particular,^12^ has been conceptualised as a key predictor of LBP and back health, as well as mediating the association between psychosocial factors and LBP and back health (figure 1), we propose several hypotheses regarding LTPA. First, we hypothesise that individuals with cLBP will differ in their physical activity patterns from individuals without cLBP. Relatedly, we expect that individuals with cLBP will experience more challenges in regulating their health behaviour (eg, lower intention and poorer self-regulation; figure 1) than participants without cLBP, for example, due to their respective pain-related and physical activity-related cognitions (eg, higher levels of kinesiophobia). Second, we hypothesise that higher average levels of LTPA will be associated with lower levels of pain and better back health, posture and mobility.

With regard to psychosocial factors, we expect that higher levels of pain-related and activity-related cognitions that can be considered risk factors for cLBP (eg, kinesiophobia) will be associated with higher pain intensity and poorer back health, whereas resources (eg, pain self-efficacy) will be associated with lower pain intensity and better back health. On the contextual variables side, we will explore whether higher levels of perceived general and pain-related social support are associated with lower pain intensity and better back health. In collaboration with the overall research consortium, we will also investigate whether and how spinal morphology, mechanics and motion are linked to LBP, back health and physical activity.

In terms of the macrolongitudinal perspective, we expect the variables measured at baseline, 3 months and 6 months after baseline to be relatively stable over the chosen time intervals, as no intervention has been implemented.

#### Within-person hypotheses

At the within-person level, we will examine how changes in psychosocial variables covary with changes in physical activity and subsequently with LBP intensity and back health. We hypothesise that people will report lower pain intensity and better back health at times when they are more physically active in their leisure time than usual. We also expect to observe lower pain intensity, better back health and more LTPA engagement than usual at times when self-control, pain self-efficacy and positive affect are higher than usual, and fear of movement and negative affect are lower than usual.^26^,^29^ We will explore these relationships at different levels of temporal resolution (eg, momentary, daily) and for different time lags (eg, same measurement time, different time points).

## Methods and analysis

### Study design

**Figure 2:**
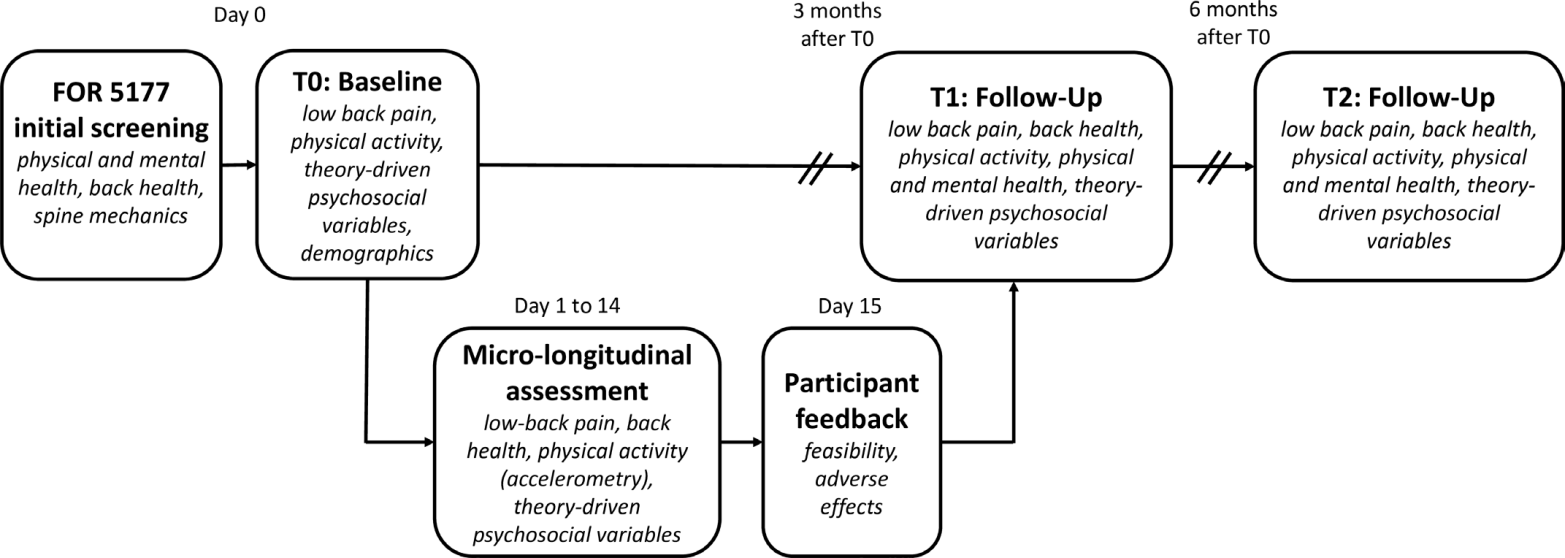
Study design: The PRIA study is a prospective macro-longitudinal study spanning 6 months, with a 14-day micro-longitudinal EMA phase. EMA, ecological momentary assessment; PRIA, Psychologie und Rückengesundheit im Alltag.

This observational study uses a prospective macro-longitudinal design spanning 6 months, complemented by a micro-longitudinal phase using EMA over a 14-day period (figure 2). The study is embedded in an interdisciplinary research consortium, FOR 5177,^30^ which is investigating spinal morphology (eg, spinal shape), mechanics (eg, lumbar spinal loading) and motion (eg, spino-pelvic kinematics and physical activity) as well as interventions and mechanisms, and how they are related to and associated with cLBP. The overall goal of the FOR 5177 research consortium is to improve the understanding of the aetiology and pathogenesis of cLBP, to propose novel strategies for patient stratification, and to advance the prevention and treatment of cLBP. To this end, static imaging (ie, magnetic resonance imaging) and short physical assessments, as they represent the current clinical diagnostic approach in cLBP, are complemented by dynamic investigations of spinal mobility and loading, and assessments of physical activity and psychosocial factors in daily life. In the following, we describe the PRIA subproject, including the specific study sample, procedures and measures that contribute to FOR 5177 and can also serve as an independent analysis sample.

### Sample and recruitment

Adults have been recruited in the greater Berlin area since January 2023. Recruitment methods include local advertising at Charité – Universitätsmedizin Berlin, Humboldt Universität zu Berlin and MSB Medical School Berlin (via postal flyers, notice boards, internet outreach and social media), outreach to the general public (via newspapers, magazines, podcasts, TV), collaboration with local companies and administrative authorities and word of mouth. In addition, the study was promoted during the Long Night of Sciences, a public science fair in Berlin in 2022, 2023 and 2024. Participants are emailed information material and invited for a brief introductory telephone call to explain the procedure, review the inclusion and exclusion criteria and schedule a baseline appointment. After receiving verbal and written information for the PRIA study, including information on the sampling scheme and study devices, participants provide written informed consent.

### Inclusion and exclusion criteria

Participants must meet the following inclusion criteria: (1) report lumbopelvic pain for the past 12 weeks or longer (ie, participants with cLBP), report intermittent lumbopelvic pain or no lumbopelvic pain at all (ie, participants without cLBP), (2) be between 18 and 64 years old, (3) be able to hear an alarm from a smartphone and (4) be able to read and understand German text on a 6-inch smartphone and a 10-inch tablet. In terms of exclusion criteria, participants were also required not to: (1) be enrolled in other ongoing clinical trials, (2) be currently pregnant, (3) be a professional, competitive or top athlete, and (4) have a BMI >28 kg/m^2^. Further exclusion criteria are listed in the FOR 5177 registration in the German Clinical Trials Register (DRKS number: DRKS00027907).

### Procedures

Participants undergo an initial screening at the Charité – Universitätsmedizin Berlin. Each participant in the PRIA subproject then completes a battery of self-administered questionnaires at baseline (time 0, T0), with subsequent follow-up questionnaires (time 1 and 2, T1 and T2; macro-longitudinal design). Data are collected via an online survey platform (Unipark, Tivian XI, Cologne, Germany) using a study tablet on-site (T0) or a personal device at home (eg, computer, tablet, smartphone; T1–T2).

Participants are then instructed in the time-sampling procedure, the use of the Android study smartphone (Nokia 6.3) equipped with the movisensXS app (movisens GmbH, Germany), and the proper handling of the accelerometer (Move 4, movisens GmbH, Germany). For the following 14 days, participants wear the activity sensor to collect data on their physical activity during waking hours. In addition, at five alarm-triggered measurement occasions per day (at 09:00, 12:00, 15:00, 18:00 and 21:00), participants fill out short self-report questionnaires using the study smartphone and the movisensXS app. Each assessment takes approx. 2 min to complete, resulting in a total of 2.5 hours of diary assessments during the entire EMA phase.

At the end of the 14-day period, participants return the study devices and are reimbursed €30 for completing the assessment. On day 16, participants are being emailed a link to a feedback questionnaire about their experience of the EMA phase. Participants then receive individualised feedback on selected EMA variables. At T1 (3 months after T0) and T2 (6 months after T0), participants are again being emailed links to the follow-up questionnaires.

### Benefits and harms

Given the non-invasive nature of both the accelerometer measurements and the online questionnaires, we do not anticipate any harm beyond the time commitment required of participants. In addition, participants were reimbursed €30 and received individualised feedback on selected variables assessed within the EMA.

### Patient and public involvement

Participants are encouraged to report any questions or adverse effects they experience during the study to the study facilitator via text message on the movisensXS app, email or phone call. After the EMA phase, participants are requested to provide feedback on the study to improve future assessments. During the study preparation phase, the researchers consulted the Patient Advisory Board on participants’ feedback and concerns.

### Measures

#### Macro-longitudinal measures

Table 1 provides an overview of the variables measured at T0, T1 and T2. At T0, participants provide information on demographic characteristics, LBP chronicity status, LBP intensity and pain-related disability, back health, physical activity and theory-based psychological variables. Time-sensitive variables are being reassessed at T1 and T2.

**Table 1:** Variables and sampling scheme for the macro-longitudinal study period

Variable	Initial screening*	Baseline (T0)	3 months (T1)	6 months (T2)
Demographic information: Age, gender, marital status, children, living situation, education, job status, income, subjective social status	–	x	–	–
Sex, occupational field, years of work, occupational and family burdens, occupational disability, retirement status	x	–	–	–
**Behavioural outcomes**				
Physical activity and sedentary behaviour: International Physical Activity Questionnaire long version	–	x	x	x
Back posture and mobility: Back Posture, Movement and Mobility self-report questionnaire, Postural Awareness Scale	–	x	x	x
Back posture and mobility (objective): Idiag M360 (Idiag AG, Switzerland)	x	–	–	–
Medication intake: Medication Quantification Scale	–	x	x	x
**Behaviour-related health outcomes with respect to the lower back**				
Low back health: Single item adapted from the SF-36/SF-12 Health Survey	x	–	x	x
Low back pain: Chronic Pain Grade Questionnaire, chronicity of LBP	x	x	x	x
Disability due to low back pain (Roland-Morris Disability Questionnaire)†	x	–	x	x
General physical and mental health: Health-related Quality of Life (SF-36^1^/SF-12 Health Survey^2^)	x^1^	–	x^2^	x^2^
Depressive symptoms and anxiety (Patient Health Questionnaire-4)	x	–	x	x
Perceived Stress Scale (PSS-10)	x	x	x	x
**Theory-based psychological variables**				
Activity-related cognitions: risk perception, outcome expectancies, self-efficacy, intention, planning	–	x	x	x
Habit strength (Self-Report Behavioural Automaticity Index)	x	–	x	x
Back-related and pain-related beliefs: Pain catastrophising (Pain Catastrophising Scale)†, pain self-efficacy (Pain Self-Efficacy Questionnaire)†, Avoidance-Endurance Fast Screen†	–	x	x	x
Fear of movement (Tampa Scale for Kinesiophobia)	x	–	x	x
Social processes: Perceived general social support; responses of others to pain (West Haven-Yale Multidimensional Pain Inventory, Part II)	–	x	x	x
Affect: Positive and Negative Affect Schedule	–	–	x	x
General self-control: Self-Control Scale	–	x	x	x

* The initial screening is not part of this study, but of another subproject within the FOR 5177 research unit—data are available.

† Questions will only be presented to participants who report to currently experience LBP.

LBP, low back pain.

#### Primary outcome

LTPA is the primary outcome variable, assessed as non-work-related moderate-to-vigorous physical activity. Participants complete an adapted version of the validated German version of the International Physical Activity Questionnaire, IPAQ long.^31 32^ The questionnaire covers different intensities of physical activity in four activity domains including housework and gardening, work-related activities, transport, LTPA and sedentary behaviour. Participants report the number of hours and/or minutes spent in moderate and vigorous activities within each activity domain over the past 7 days. The IPAQ data will be processed according to the IPAQ protocol.^33^ The validity of the IPAQ will be evaluated empirically by assessing its agreement with the accelerometer data collected in this study.

#### Secondary outcomes

Secondary outcome variables encompass behaviour-related variables related to lower back, such as LBP intensity, back health, back posture and mobility, sedentary behaviour and pain medication.

##### Behaviour-related health outcomes

To assess LBP intensity and pain-related disability*,* we use the Chronic Pain Grade Questionnaire (CPGQ),^34 35^ with instructions adapted to focus on LBP. Chronicity of LBP is assessed using a binary (yes/no) item including the criterion of constant or almost daily LBP in the previous 12 weeks.^2^

As an indicator of back health, we adapted a single item from the SF-36,^36^ which assesses subjective health: ‘How would you rate the health of your lower back?’, with a 5-point Likert scale of 1 (poor), 2 (not so good), 3 (good), 4 (very good), and 5 (excellent).

General health-related quality of life is assessed using the German SF-12 Health Survey (SF-12).^36^ Perceived stress is measured with the validated German version of the 10-item Perceived Stress Scale (PSS-10).^37 38^

##### Behavioural outcomes

To measure back posture and mobility, we use an adapted version of the Back Posture, Movement and Mobility (B-PMM) self-report questionnaire.^16^ The B-PMM self-report questionnaire consists of 13 items that assess perceived *back posture* (eg, ‘When I work, I pay attention to a posture that is gentle on my back.’), perceived back movement (eg, ‘When I am active during my leisure time, my back is also in a lot of movement (eg, twisting, bending, lifting).’) and back mobility (‘I am very flexible in the back.’). Participants can respond to the items using a 6-point Likert scale, ranging from 1 (not true at all) to 6 (extremely true). For posture and movement, the mean score is calculated from the relevant items. The validity of the B-PMM will be tested by determining the agreement with the Postural Awareness Scale (PAS),^39^ an established scale to assess the awareness of body posture in patients with chronic pain and used in the PRIA study, and with objective back measurement data from other subprojects of the research consortium.

We measure sedentary behaviour at work and during leisure time in the past 7 days by self-report using three items adapted from the IPAQ long.^31 32^ Finally, to assess pain medication, we use the Medication Quantification Scale (MQS).^40^ We record drug name, dosage (in milligrams), form (ie, pill, patch, balm, suppository, other), intake as needed or regular, intake time of day (ie, in the morning, at noon, in the afternoon, in the evening), pain localisation (ie, LBP, neck pain, headache, other), side effects (ie, nausea, vomiting, dry mouth, dizziness, sweating, tiredness, disorientation, concentration problems, stomach upset, other), and further comments, separately for each drug. The drug names will be categorised into medication subclasses, for which risk weights are available.^41^ This information can be used to calculate daily oral morphine equivalents.^42^ The relative dosage scores will be individually calculated by comparing the participant’s daily dosage with the physicians’ desk reference (current year) recommendations (eg,^43^). Thereby, three aspects of medications (drug class, dosage, risk) can be quantified.

### Determinants of behavioural and behaviour-related health outcomes

We measure (a) cognitions specific to LTPA, (b) cognitions specific to pain perception regarding the self and the social environment and (c) general cognitions and affect.

#### Activity-related cognitions

Based on the HAPA,^19^ we assess risk perception with an adapted 4-item scale introduced by Lippke *et al*,^44^ outcome expectancies with an adapted 8-item scale based on the measurement by Meng *et al*,^45^ intention to be physically active, physical activity-related self-efficacy and action planning based on the measures implemented by Sniehotta *et al*^46^ and Knoll *et al*^47^ with several adaptations (eg, for intention, the time reference is to the next 7 days instead of the last 7 days). Habit strength is measured by the Self-Report Behavioural Automaticity Index.^48^

#### Back-related and pain-related cognitions

Based on the fear-avoidance model,^49 50^ we assess pain-related cognitions, pain catastrophising using the validated German version of the Pain Catastrophising Scale,^51^ pain self-efficacy using the validated German version of the Pain Self-Efficacy Questionnaire (PSEQ),^52^ the FESS (Fragebogen zur Erfassung der schmerzspezfischen Selbstwirksameit),^53^ and fear-avoidance and endurance-related responses to pain using the Avoidance-Endurance Fast Screen.^54^

#### Behaviour-unspecific, general cognitions and affect

General self-control is measured using the validated German version of the Self-Control Scale.^55^,^57^ We also measure perceived general social support using the short form of the Social Support Questionnaire.^58^ We assess positive affect and negative affect using 12 items from the short form of the Positive and Negative Affect Schedule (short form PANAS^59^, based on PANAS^60^) in a German translation by Krohne *et al*^61^ (translation of one item adapted).

#### Social factors

We assess responses of significant others to pain reported by participants using the validated German version of the West Haven-Yale Multidimensional Pain Inventory (WHYMPI/MPI), Part II^62^ (Patienten MPI-D^63^). Part II of the WHYMPI/MPI questionnaire consists of three subscales that capture solicitous, distracting and punishing responses of a significant other (eg, spouse) while participants display pain behaviours.

### Micro-longitudinal measures

The variables collected during the EMA study phase correspond to the variables in the macro-longitudinal study. The sampling schedule and the wording of the items used in the EMA study can be found in online supplemental table 1.

#### Physical activity

Physical activity is assessed both objectively using accelerometer data and through self-report. Objective data are collected passively during waking hours using the Move 4 activity sensor.^64^ The sensor, worn on the right hip, continuously records data using a 3D accelerometer, gyroscope, barometric pressure and temperature. The device has no display and does not provide real-time feedback on activity. Combined with movisens’ DataAnalyzer analysis software, the collected data are used to calculate activity class, body position, steps, energy expenditure and metabolic equivalent of task. As part of the data cleaning process, the recording time is adjusted using the movisens’ UnisensViewer software to represent exactly 14 study days. The daily wear time is determined by subtracting the non-wear time from the total recording time. According to the guidelines of Troiano *et al*,^65^ days with 10 hours or more of wear time are considered valid.

Additionally, on each of five measurement occasions per day, participants are asked to indicate (a) whether they were physically active in their leisure time (ie, non-work-related) for at least 10 consecutive minutes, (b) the type of activity and (c) the exact times of the activity for up to two activity types per measurement occasion.

#### Low back pain

At each measurement occasion, we assess the occurrence of LBP with the binary item: ‘Since the last questionnaire, have you had or are you currently experiencing low back pain?’ (yes/no). If pain has occurred, participants report the intensity of their LBP using a single item adapted from the CPGQ^34 35^: ‘How would you rate your low back pain since the last questionnaire?’, on an 11-point scale ranging from 0 (no pain) to 10 (pain as bad as it could be). Each evening (ie, at 21:00), participants also rate the most intense LBP of the day from 0 (no pain) to 10 (pain as bad as it could be).

#### Low back health

At each measurement, we ask participants to rate their back health using a single item adapted from the SF-36^36^: ‘How has your lower back felt since the last questionnaire?’, using a 5-point Likert scale of 1 (poor), 2 (not so good), 3 (good), 4 (very good) and 5 (excellent).

#### Physical activity-related cognitions

We assess physical activity-related self-efficacy by asking: ‘At the moment, how confident are you that you can manage to be physically active in your leisure time, even if you find it difficult?’, and physical activity-related intention strength with the item: ‘At the moment, to what extent do you intend to be physically active in your leisure time?’. Both items are rated on a 10-point scale with anchors at 1 (not at all) and 9 (very much) and are based on research by Inauen *et al*.^23^ For each reported physical activity, participants also indicated whether their activity was planned or spontaneous using two items: ‘I planned this activity in advance’ and ‘I was spontaneously physically active’, and the 6-point Likert scale from 1 (I completely disagree) to 6 (I completely agree).

#### Pain-related cognitions

If participants report the occurrence of pain since the last questionnaire, we assess kinesiophobia as a potential pain-related risk factor for physical inactivity. To do this, we use a single item adapted from the Tampa Scale for Kinesiophobia^66^: ‘At the moment, I’m afraid that I might injure myself if I exercise’, rated on a scale from 1 (I completely disagree) to 6 (I completely agree). On the resource side, we assess pain self-efficacy with a single item adapted from the PSEQ^52^ (FESS^53^): ‘At the moment, I can still do things that I enjoy doing, such as hobbies or leisure activity, despite pain’, scored on the same response scale as above.

#### Pain medication

Based on the MQS,^40^ participants are asked at each measurement occasion whether they had taken any medication since the last questionnaire (yes/no). If yes, participants are asked to provide the drug name, dosage (in milligrams), form (ie, pill, patch, balm, suppository, other) and pain localisation (ie, low back pain, upper back pain, neck pain, headache, other), separately for up to four medications per measurement. In the evening (ie, at 21:00), participants report whether any side effects occurred that day.

#### Social factors

If pain has occurred, participants are asked if they have received support from other people related to their LBP since the last questionnaire (yes/no). If participants report having received support, they are asked who the support provider was (partner, family member, friend, professional caregiver, other). Furthermore, we assess received instrumental support as joint problem solving such as discussing the problem with one’s partner and taking concrete actions^67 68^ with the item: ‘The person discussed possible solutions with me or did something about the problem’, and received emotional support with the item ‘The person comforted me or hugged me’,^69^ both on a scale from 1 (I completely disagree) to 6 (I completely agree).

#### Affect, perceived stress and self-control

At the beginning of each measurement, we assess positive and negative affect by asking ‘How … do you feel at the moment?’, presenting five single positive affect items (inspired, alert, excited, enthusiastic, determined) and six negative affect items (afraid, upset, nervous, distressed, jittery, annoyed). The affect items are rated on a scale of 1 (very slightly or not at all), 2 (a little), 3 (moderately), 4 (quite a bit) and 5 (very much). The items are derived from the short form of the PANAS^59 61^ (translation of one item adapted). Using the same item format and response scale, we additionally ask participants to rate their perceived stress. Next, we measure self-control using one positively and one negatively framed item adapted from the State Self-Control Capacity Scale (SMS-5)^70^: ‘At the moment, I feel like I have no willpower left’ and ‘At the moment, I feel balanced’. Both items are rated on a scale from 1 (fully disagree) to 7 (fully agree).

#### Generalisability

At 21:00, participants report whether they worked that day and, if so, the exact hours they worked. Participants then report how typical their day was using the item ‘Now think about your usual activities on a typical weekday or weekend day like today. Was today typical for your everyday life?’, with a 5-point Likert scale of 1 (not at all or very little), 2 (a little), 3 (to some extent), 4 (a lot) and 5 (extremely). If the answer is ‘to some extent’ or lower, participants are asked to provide additional information on that day. First, with the multiple-choice item ‘What made today less typical than usual?’, with the response format 1 (less low back pain than usual), 2 (more low back pain than usual), 3 (less physical activity than usual), 4 (more physical activity than usual), 5 (other peculiarities). Second, with the open-ended question ‘What was different from usual?’.

### Power analysis

To test the micro-longitudinal hypotheses, an a priori power calculation for the LTPA outcome indicated that over n=2000 individuals (ie, number of individuals required) and at least n=14 (ie, number of repeated measures) would be required to detect small-sized population effects (given the unknown size of the cross-level interaction which is typically smaller than a main effect,^71^) with a power of ≥0.95. We aim to achieve this, but more realistically, assuming a power of ≥0.80, a sample size of at least n=200 (accounting for 30% attrition) is required to detect small-sized population effects.^72^

### Data analyses

Statistical analyses will be carried out both throughout the data collection process for monitoring purposes and at the end of the data collection. Descriptive statistics will be calculated for all variables (see table 1, online supplemental table 1), together with plausibility checks, assessment of distribution characteristics, identification of outliers and analysis of missing data.

#### Between-person hypotheses

The relationship between physical activity and behaviour-related health outcomes will be evaluated through regression models. Additionally, path analyses will be employed to test our hypotheses regarding longitudinal direct and indirect associations between theory-based psychological variables and LTPA (figure 1).

#### Within-person hypotheses

Intraclass correlation coefficients, Pearson correlations, as well as repeated measures correlations,^73^ will be calculated. To account for the nested structure of the micro-longitudinal data, multilevel models will be calculated with the primary outcome (ie, LTPA) and the secondary outcomes (eg, LBP intensity, back health) as dependent variables. Based on the BHBM (figure 1), psychosocial variables will be included as level-1 predictors. LBP chronicity status (ie, participants with and without cLBP) will be considered a person-level predictor, moderating associations between within-person predictors and outcomes. We will specify multilevel models with fixed and random effects. Hypothesis testing for the fixed effects will provide insights into the relationships between LBP intensity, back health, LTPA and its within-person and between-person predictors. To test the theoretical assumptions (figure 1), we will specify within-person mediation models and estimate indirect effects. In collaboration with the research consortium, we will also investigate whether the association between LTPA and its predictors is of different size depending on, for example, sex, spinal morphology and mechanics.

### Ethics and dissemination

The university’s ethics committee at the MSB Medical School Berlin approved the study on 8 March 2021 (approval number MSB-2021/59, amendment approved on 10 November 2023, amendment number MSB-2023/145). Ethical approval for the initial screening of FOR 5177 was granted by Charité – Universitätsmedizin Berlin (EA1/058/21). Written informed consent is obtained from each participant prior to enrolment. The PRIA study is funded by the German Research Foundation (DFG; project number 439742772, grant numbers FL 879/2-1, STE 477/22-1, PU 762/1-1, SCHM 2572/11-1 and SCHM 2572/13-1). The study is registered in the German Clinical Trials Register (DRKS00032978, registration date:22 December 2022) and is also listed on the International Clinical Trials Registry Platform. Data collection started on 9 January 2023 and the planned study end date is 6 January 2026. Data storage is in accordance with the EU General Data Protection Regulation, with all data pseudonymised and securely stored on a server at MSB Medical School Berlin.

The results of this research will be reported to the funding agency (DFG), published in peer-reviewed international journals and presented at national and international conferences. In addition, wider dissemination to the public is planned through events such as the Long Night of Sciences (a public science fair) in Berlin and various media channels. Findings based on a subset of the self-reported EMA data have been published previously.^74^

## Discussion

As part of a larger interdisciplinary research consortium, the PRIA study investigates diagnostic, preventive and therapeutic aspects of cLBP. It complements the current clinical diagnostic approach to cLBP by examining LBP intensity and back health in participants with and without cLBP in their everyday lives. In doing so, the study implements methods that go beyond cross-sectional analyses of data collected at a single point in time. The results of this study are expected to contribute to the development of improved diagnostics, for example, by looking more closely at how physical activity and LBP intensity unfold in the daily lives of people with cLBP,^22 75^ and effective, personalised interventions by addressing risk factors and resources for cLBP and back health in daily life. One promising avenue of prevention research is just-in-time adaptive interventions using mobile technology.^76^ Research that improves our understanding of the subtle connections between pain and physical activity can help shape psychotherapeutic and behavioural recommendations, potentially improving the health, longevity and quality of life for people with chronic pain.

For the prevention of cLBP, individuals are expected to be able to actively minimise psychosocial risk factors and build on resources, such as engaging in physical activity. In the context of cLBP, the latter may encompass both LTPA^11 12^ and adjustment and modification of back posture.^39^ Currently, there is no consistent evidence on which types of physical activity (eg, in terms of intensity, duration and frequency of LTPA^13^) are most beneficial for people with cLBP to reduce pain intensity and promote back health, and evidence as to how the associations between pain and physical activity unfold at the momentary level.^75^ A deeper understanding of the associations between cLBP, different aspects of physical activity and their theory-based psychological predictors is needed to guide future recommendations and interventions. With its comprehensive approach combining theory, evidence and assessment methods from health and pain psychology, but also drawing on insights from behavioural medicine, biomechanics and movement sciences, the PRIA intensive longitudinal study aims to advance the field of cLBP prevention and treatment.

### Strengths, limitations and challenges

A notable advance of the PRIA study, in line with previous studies in primary prevention of chronic pain,^16^ is its shift from a pathological perspective to a more resource-oriented approach to understanding cLBP.^77^ While previous research has focused primarily on examining modifiable risk factors such as health risk behaviours (eg, heavy lifting, prolonged sitting) and negative health-related cognitions (eg, fear of movement), the PRIA study broadens this scope. It examines these risk factors alongside theory-based, modifiable resources, including physical activity and self-efficacy, providing a more balanced view of cLBP prevention and management. A unique value of the PRIA study is that it examines psychosocial risk factors and resources in a cohort of individuals with and without cLBP. This will allow us to examine not only the role of resources and risk factors in the presence of pain (ie, cohort of people with cLBP), but also the role of resources and risk factors in promoting back health (ie, cohort of asymptomatic individuals). This approach will provide valuable insights for the development of effective behavioural interventions aimed at both the prevention of LBP and the promotion of back health.

In addition to a traditional macro-longitudinal approach, the PRIA study uses an intensive longitudinal design that involves multiple sampling of participants’ momentary experiences and behaviours in their everyday environments, rather than relying on isolated laboratory-based assessments. This allows for high temporal data resolution at the intra-individual level, providing new theoretical insights into the within-person dynamics of psychosocial and behavioural risks and resources associated with LBP over time in everyday life.^78^ However, this approach also presents challenges, such as the potential for higher dropout rates and increased participant burden. To mitigate these risks, the study includes financial incentives and individualised feedback to improve participant retention and engagement. Another challenge may be the potential adverse effect of repeated pain assessments, which could increase the perception of pain by focusing the participant’s attention on adverse bodily experiences. However, recent research supports the usability and feasibility of EMA on a smartphone to collect real-time data on cLBP.^79^ Importantly, the assessment of pain using smartphones does not appear to have a negative impact on pain intensity trajectories.^15^

A key strength of the PRIA study is its multimethod approach to assessing physical activity, including both objective measures and self-reports. By using objective data on physical activity, the study helps to mitigate problems commonly associated with method variance, such as inflated correlations between self-reported predictors and self-reported outcomes. In addition, the use of accelerometry reduces the potential for overestimation or underestimation of physical activity. However, the use of accelerometers has limitations, including the potential for missing or unusable data, participant non-compliance with wearing the device and technical malfunction. A major disadvantage of accelerometers is their inability to capture detailed information about the specific type of physical activity (eg, yoga, walking). To address this, the PRIA study combines accelerometer data with self-reported measures of physical activity.

Due to its observational design, this study does not permit causal conclusions to be drawn. Instead, the study aims to highlight key risk factors and resources for cLBP that should be examined further in interventional studies. For example, the findings on the temporal dynamics of pain and modifiable resources and risk factors are expected to inform the development of mobile technology-based interventions (eg, just-in-time adaptive interventions^76^) that support behavioural approaches to both LBP treatment and back health promotion. Despite the potential methodological challenges and limitations, the theoretical insights gained from our observational study are expected to improve the understanding of risk factors and protective resources related to cLBP.

## Supplementary material

10.1136/bmjopen-2025-109887 online supplemental file 1

## References

[1] Ferreira ML, de Luca K, Haile LM (2023). Global, regional, and national burden of low back pain, 1990–2020, its attributable risk factors, and projections to 2050: a systematic analysis of the Global Burden of Disease Study 2021. Lancet Rheumatol.

[2] von der Lippe E, Krause L, Porst M (2021). Prevalence of back and neck pain in Germany. Results from the BURDEN 2020 Burden of Disease Study. *J Health Monit*.

[3] Grobe T, Bessel S (2023). Gesundheitsreport: Arbeitsunfähigkeiten. Aqua-Institut für angewandte Qualitätsförderung und Forschung im Gesundheitswesen GmbH.

[4] Nieminen LK, Pyysalo LM, Kankaanpää MJ (2021). Prognostic factors for pain chronicity in low back pain: a systematic review. Pain Rep.

[5] Waddell G (1987). 1987 Volvo Award in Clinical Sciences: A New Clinical Model for the Treatment of Low-Back Pain. Spine (Phila Pa 1986).

[6] Eich W, Diezemann-Prößdorf A, Hasenbring M (2023). Psychosocial factors in pain and pain management : A statement. Schmerz.

[7] Levenig CG, Hasenbring MI, Günnewig L (2024). Treatment expectations: You get what you expect and depression plays a role. J Pain.

[8] Hasenbring MI, Levenig C, Hallner D (2018). Psychosocial risk factors for chronic back pain in the general population and in competitive sports : From theory to clinical screening-a review from the MiSpEx network. Schmerz.

[9] López-Bueno R, Ahmadi M, Stamatakis E (2023). Prospective associations of different combinations of aerobic and muscle-strengthening activity with all-cause, cardiovascular, and cancer mortality. JAMA Intern Med.

[10] World Health Organisation (2020). World Health Organization 2020 guidelines on physical activity and sedentary behaviour. 2020 dec. report no.: 0306-3674 (print) contract no.: 24.

[11] Zhu Y, Zhang H, Li Q (2024). Association of aerobic and muscle-strengthening activity with chronic low back pain: population-based study. Spine J.

[12] Gupta N, Rasmussen CL, Hartvigsen J (2022). Physical activity advice for prevention and rehabilitation of low back pain- same or different? A study on device-measured physical activity and register-based sickness absence. J Occup Rehabil.

[13] Comachio J, Ferreira ML, Mork PJ (2024). Clinical guidelines are silent on the recommendation of physical activity and exercise therapy for low back pain: A systematic review. J Sci Med Sport.

[14] Antonovsky A, Liamputtong P (2024). Handbook of Concepts in Health, Health Behavior and Environmental Health.

[15] Gruszka P, Stammen C, Bissantz N (2019). Pain vs. comfort diary: A fully remote app-based experiment. Eur J Pain.

[16] Wilhelm LO, Lederle N, Diering L-E (2025). Linking physical activity to workers’ low back pain, back health, and theory-based psychological variables: study protocol of the workHealth intensive longitudinal observational study. BMC Public Health.

[17] Vlaeyen JWS, Linton SJ (2000). Fear-avoidance and its consequences in chronic musculoskeletal pain: a state of the art. Pain.

[18] Hasenbring MI, Chehadi O, Titze C (2014). Fear and anxiety in the transition from acute to chronic pain: there is evidence for endurance besides avoidance. Pain Manag.

[19] Schwarzer R (2008). Modeling health behavior change: How to predict and modify the adoption and maintenance of health behaviors. Applied Psychology.

[20] Jekauc D, Gürdere C, Englert C (2024). The contribution and interplay of implicit and explicit processes on physical activity behavior: empirical testing of the physical activity adoption and maintenance (PAAM) model. BMC Public Health.

[21] Huijnen IPJ, Verbunt JA, Roelofs J (2009). The disabling role of fluctuations in physical activity in patients with chronic low back pain. Eur J Pain.

[22] May M, Junghaenel DU, Ono M (2018). Ecological momentary assessment methodology in chronic pain research: A systematic review. J Pain.

[23] Inauen J, Shrout PE, Bolger N (2016). Mind the gap? An intensive longitudinal study of between-person and within-person intention-behavior relations. Ann Behav Med.

[24] Keogh E (2022). Sex and gender differences in pain: past, present, and future. Pain.

[25] Keogh E, Boerner KE (2024). Challenges with embedding an integrated sex and gender perspective into pain research: Recommendations and opportunities. Brain Behav Immun.

[26] Chenot J-F, Greitemann B, Kladny B (2017). Non-Specific Low Back Pain. Dtsch Arztebl Int.

[27] Costal L da CM, Maherl CG, McAuleyl JH (2011). Self‐efficacy is more important than fear of movement in mediating the relationship between pain and disability in chronic low back pain. Eur J Pain.

[28] Kichline T, Cushing CC, Connelly M (2022). Microtemporal relationships in the fear avoidance model: An ecological momentary assessment study. Clin J Pain.

[29] Fjeld MK, Årnes AP, Engdahl B (2023). Consistent pattern between physical activity measures and chronic pain levels: the Tromsø Study 2015 to 2016. Pain.

[30] Gepris The Dynamics of the Spine: Mechanics, Morphology and Motion towards a comprehensive Diagnosis of Low Back Pain n.d.

[31] Wanner M, Probst-Hensch N, Kriemler S (2016). Validation of the long international physical activity questionnaire: Influence of age and language region. Prev Med Rep.

[32] Craig CL, Marshall AL, Sjöström M (2003). International physical activity questionnaire: 12-country reliability and validity. Med Sci Sports Exerc.

[33] International Physical Activity Questionnaire team (2005). Guidelines for data processing and analysis of the international physical activity questionnaire (ipaq) – short and long forms.

[34] Von Korff M, Ormel J, Keefe FJ (1992). Grading the severity of chronic pain. J Pain.

[35] Klasen BW, Hallner D, Schaub C (2004). Validation and reliability of the German version of the Chronic Pain Grade questionnaire in primary care back pain patients. Psychosoc Med.

[36] Morfeld M, Kirchberger I, Bullinger M (2011). SF-36. Fragebogen Zum.

[37] Cohen S, Kamarck T, Mermelstein R (1983). A global measure of perceived stress. J Health Soc Behav.

[38] Klein EM, Brähler E, Dreier M (2016). The German version of the Perceived Stress Scale - psychometric characteristics in a representative German community sample. BMC Psychiatry.

[39] Cramer H, Mehling WE, Saha FJ (2018). Postural Awareness Scale. APA PsycTests.

[40] Harden RN, Weinland SR, Remble TA (2005). Medication Quantification Scale Version III: Update in medication classes and revised detriment weights by survey of American Pain Society physicians. J Pain.

[41] De Clifford-Faugère G, Nguena Nguefack HL, Godbout-Parent M (2024). The Medication Quantification Scale 4.0: An updated index based on prescribers’ perceptions of the risk associated with chronic pain medications. J Pain.

[42] Nielsen S, Degenhardt L, Hoban B (2016). A synthesis of oral morphine equivalents (OME) for opioid utilisation studies. Pharmacoepidemiol Drug Saf.

[43] Dowell D, Ragan KR, Jones CM (2022). CDC Clinical Practice Guideline for Prescribing Opioids for Pain - United States, 2022. MMWR Recomm Rep.

[44] Lippke S, Ziegelmann JP, Schwarzer R (2004). Behavioral intentions and action plans promote physical exercise: A longitudinal study with orthopedic rehabilitation patients. J Sport Exerc Psychol.

[45] Meng K, Seekatz B, Roßband H (2009). Entwicklung eines standardisierten Rückenschulungsprogramms für die orthopädische Rehabilitation. Rehabilitation.

[46] Sniehotta FF, Schwarzer R, Scholz U (2005). Action planning and coping planning for long-term lifestyle change: theory and assessment. Eur J Soc Psychol.

[47] Knoll N, Hohl DH, Keller J (2017). Effects of dyadic planning on physical activity in couples: A randomized controlled trial. Health Psychol.

[48] Gardner B, Abraham C, Lally P (2012). Towards parsimony in habit measurement: testing the convergent and predictive validity of an automaticity subscale of the Self-Report Habit Index. Int J Behav Nutr Phys Act.

[49] Hasenbring MI, Hallner D, Rusu AC (2009). Fear-avoidance- and endurance-related responses to pain: development and validation of the Avoidance-Endurance Questionnaire (AEQ). Eur J Pain.

[50] Vlaeyen JWS, Crombez G, Linton SJ (2016). The fear-avoidance model of pain. Pain.

[51] Sullivan MJL, Bishop SR, Pivik J (1995). The Pain Catastrophizing Scale: Development and validation. Psychol Assess.

[52] Nicholas MK, McGuire BE, Asghari A (2015). A 2-item short form of the Pain Self-efficacy Questionnaire: development and psychometric evaluation of PSEQ-2. J Pain.

[53] Mangels M, Schwarz S, Sohr G (2009). Der Fragebogen zur Erfassung der schmerzspezifischen Selbstwirksamkeit (FESS): Eine Adaptation des Pain Self-Efficacy Questionnaire für den deutschen Sprachraum. Diagnostica.

[54] Wolff SV, Willburger R, Hallner D (2018). Avoidance-Endurance Fast-Screen (AE-FS). Schmerz.

[55] Tangney JP, Baumeister RF, Boone AL (2004). High self-control predicts good adjustment, less pathology, better grades, and interpersonal success. J Pers.

[56] Bertrams A, Dickhäuser O (2009). Messung dispositioneller Selbstkontroll-Kapazität: Eine deutsche Adaptation der Kurzform der Self-Control Scale (SCS-K-D). Hogrefe Verlag.

[57] Singh RK (2022). Normierung und testtheoretische Überprüfung der deutschen Adaptation der Kurzform der Self-Control Scale (SCS-K-D). Diagnostica.

[58] Fydrich T, Sommer G (2007). F-SozU: Fragebogen Zur Sozialen Unterstützung.

[59] Mackinnon A, Jorm AF, Christensen H (1999). A short form of the Positive and Negative Affect Schedule: evaluation of factorial validity and invariance across demographic variables in a community sample. Pers Individ Dif.

[60] Watson D, Clark LA, Tellegen A (1988). Development and validation of brief measures of positive and negative affect: the PANAS scales. J Pers Soc Psychol.

[61] Krohne HW, Egloff B, Kohlmann C-W (1996). Untersuchungen mit einer deutschen Version der "Positive and negative Affect Schedule (PANAS). Diagnostica.

[62] Kerns RD, Turk DC, Rudy TE (1985). The West Haven-Yale Multidimensional Pain Inventory (WHYMPI). Pain.

[63] Flor H, Rudy TE, Birbaumer N (1990). The applicability of the West Haven-Yale multidimensional pain inventory in German-speaking countries. Data on the reliability and validity of the MPI-D. Schmerz.

[64] movisens GmbH Move 4 - Activity Sensor.

[65] Troiano RP, Berrigan D, Dodd KW (2008). Physical activity in the United States measured by accelerometer. Med Sci Sports Exerc.

[66] Rusu AC, Kreddig N, Hallner D (2014). Fear of movement/(Re)injury in low back pain: confirmatory validation of a German version of the Tampa Scale for Kinesiophobia. BMC Musculoskelet Disord.

[67] Hoppmann CA, Blanchard-Fields F (2011). Problem-solving variability in older spouses: how is it linked to problem-, person-, and couple-characteristics?. Psychol Aging.

[68] Cutrona CE, Russell DW, Sarason BR, Sarason IG, Pierce GR (1990). Social support: An interactional view.

[69] Ditzen B, Hoppmann C, Klumb P (2008). Positive couple interactions and daily cortisol: on the stress-protecting role of intimacy. Psychosom Med.

[70] Lindner C, Lindner MA, Retelsdorf J (2019). Measuring self-control depletion in achievement situations: A validation of the 5-item brief State Self-Control Capacity Scale. Diagnostica.

[71] Sommet N, Weissman DL, Cheutin N (2023). How many participants do I need to test an interaction? Conducting an appropriate power analysis and achieving sufficient power to detect an interaction. Adv Methods Pract Psychol. Sci.

[72] Arend MG, Schäfer T (2019). Statistical power in two-level models: A tutorial based on Monte Carlo simulation. Psychol Methods.

[73] Bakdash JZ, Marusich LR (2017). Repeated Measures Correlation. Front Psychol.

[74] Kolodziejczak-Krupp K, Wilhelm LO, Diering L-E (2025). Psychological risk factors and resources for low back pain intensity and back health in daily life: An ecological momentary assessment study. Appl Psychol Health Well Being.

[75] Tynan M, Virzi N, Wooldridge JS (2024). Examining the association between objective physical activity and momentary pain: A Systematic review of studies using ambulatory assessment. J Pain.

[76] Nahum-Shani I, Smith SN, Spring BJ (2018). Just-in-Time Adaptive Interventions (JITAIs) in mobile health: Key components and design principles for ongoing health behavior support. Ann Behav Med.

[77] Rolli Salathé C, Elfering A (2013). A Health- and Resource-Oriented Perspective on NSLBP. ISRN Pain.

[78] Treede R-D, Rief W, Barke A (2019). Chronic pain as a symptom or a disease: the IASP Classification of Chronic Pain for the International Classification of Diseases (ICD-11). Pain.

[79] Lin WC, Burke L, Schlenk EA (2019). Use of an ecological momentary assessment application to assess the effects of auricular point acupressure for chronic low back pain. Comput Inform Nurs.

