# Supplementary material for: Physical activity and psychosocial characteristics of individuals with and without chronic low back pain in daily life: protocol for the PRIA intensive longitudinal study
Source: BMJ Open. 2025 Nov 29;15(11):e109887. doi: 10.1136/bmjopen-2025-109887 (PMC12666212; doi:10.1136/bmjopen-2025-109887)
Supplement: online supplemental file 1 [file bmjopen-15-11-s001.docx]

**ONLINE SUPPLEMENT**

**Physical activity and psychosocial characteristics of individuals with and without chronic low back pain in daily life: Protocol for the PRIA intensive longitudinal study**

Karolina Kolodziejczak-Krupp^1^, Valerie Zipper^1^, Lea O. Wilhelm^1, 2^, Lara Thiel^1^,

Christoph Stein^3^, Thomas Schäfer^4^, Matthias Pumberger^5^, Hendrik Schmidt^6^, and Lena Fleig^1^

^1^Department of Psychology, MSB Medical School Berlin, Berlin, Germany

^2^Department of Education and Psychology, Freie Universität Berlin, Berlin, Germany

^3^Department of Anaesthesiology and Intensive Care Medicine, Charité – Universitätsmedizin Berlin, Campus Benjamin Franklin, Berlin, Germany

^4^Department of Psychology, HMU Health and Medical University Erfurt, Erfurt, Germany

^5^Center for Musculoskeletal Surgery, Charité – Universitätsmedizin Berlin, Berlin, Germany

^6^Julius Wolff Institute, Berlin Institute of Health at Charité – Universitätsmedizin Berlin, Berlin, Germany

**Author Note**

Corresponding author: Lena Fleig, PhD, Department of Psychology, MSB Medical School Berlin, Rüdesheimer Str. 50, 14197 Berlin, Germany. Email: [lena.fleig@medicalschool-berlin.de](mailto:lena.fleig@medicalschool-berlin.de).

**Table S1**

*Variables and Sampling Scheme from the Micro-Longitudinal Study Period*

| **Domain** | **Item** | **Temporal reference** | **Filter design** | **9 a.m.** | **12 p.m.** | **3 p.m.** | **6 p.m.** | **9 p.m.** |
| --- | --- | --- | --- | --- | --- | --- | --- | --- |
| **Behavior** |  |  |  |  |  |  |  |  |
| Leisure-time physical activity | Since the last questionnaire, have you been physically active in your leisure time for at least 10 minutes at a time? *0 (no) – 1 (yes)* | Since the last questionnaire |  | x | x | x | x | x |
|  | What type of activity was it? *Open answer format*  When did you start? *(hh:mm)*  When did you stop? *(hh:mm)*  Another activity? *0 (no) – 1 (yes)* | Since the last questionnaire | Only if Leisure-time physical activity: yes | x | x | x | x | x |
| Plan enactment | I had planned this activity in advance.  *1 (I completely disagree) – 2 (I rather disagree) – 3 (I slightly disagree) – 4 (I rather agree) – 5 (I completely agree)*  I was spontaneously physically active.  *1 (I completely disagree) – 2 (I rather disagree) – 3 (I slightly disagree) – 4 (I rather agree) – 5 (I completely agree)* |  | Only if Leisure-time physical activity: yes | x | x | x | x | x |
| Accelerometry-based assessment of physical activity and sedentary behavior |  |  |  | Passive, continuous assessment during waking hours | | | | |
| Pain medication intake | Since the last questionnaire, have you taken any pain medication? *0 (no) – 1 (yes)* | Since the last questionnaire |  | x | x | x | x | x |
|  | Drug name *Open answer format*  Dosage (in milligrams) *Open answer format*  Form *1 (Pill) – 2 (Patch) – 3 (Balm) – 4 (Suppository) – 5 (Other)*  Pain localization *1 (Low back pain) – 2 (Upper back pain) – 3 (Neck) – 4 (Headache) – 5 (Other)*  Another drug? *0 (no) – 1 (yes)* | Since the last questionnaire | Only if Pain-medication intake: yes | x | x | x | x | x |
| Side effects | If you took any pain medication today, have any side effects occurred? *0 (no) – 1 (yes) – 2 (no pain medication intake)*  Which side effects occurred? *Open answer format* | Today | Which side effects: Only if Side effects: yes |  |  |  |  | x |
| **Behavior-related health outcomes** |  |  |  |  |  |  |  |  |
| Subjective health in relation to lower back | How has your lower back felt since the last questionnaire?  *1 (poor) – 2 (not so good) – 3 (good) – 4 (very good) – 5 (excellent)* | Since the last questionnaire |  | x | x | x | x | x |
| Low back pain | Since the last questionnaire, have you had or are you currently experiencing low back pain?  *0 (no) – 1 (yes)* | Since the last questionnaire |  | x | x | x | x | x |
| Low back pain intensity | How would you rate your low back pain since the last questionnaire?  *0 (no pain) – 10 (pain as bad as it could be)* | Since the last questionnaire | Only if low back pain: yes | x | x | x | x | x |
| Low back pain intensity | How would you rate your most intense low back pain today?  *0 (no pain) – 10 (pain as bad as it could be)* | Today |  |  |  |  |  | x |
| Perceived stress | How stressed do you feel at the moment?  *1 (very slightly or not at all) – 2 (a little) – 3 (moderately) – 4 (quite a bit) – 5 (very much)* | At the moment |  | x | x | x | x | x |
| **Theory-based psychological variables** |  |  |  |  |  |  |  |  |
| **Activity-related cognitions** |  |  |  |  |  |  |  |  |
| Intention | At the moment, to what extent do you intend to be physically active in your leisure time?  *1 (not at all) – 9 (very much)* | At the moment |  | x | x | x | x | x |
| Self-efficacy | At the moment, how confident are you that you can manage to be physically active in your leisure time, even if you find it difficult?  *1 (not at all) – 9 (very much)* | At the moment |  | x | x | x | x | x |
| **Pain-related cognitions** |  |  |  |  |  |  |  |  |
| Fear of movement | At the moment, I’m afraid that I might injure myself if I am physically active  *1 (I completely disagree) – 2 (I rather disagree) – 3 (I slightly disagree) – 4 (I rather agree) – 5 (I completely agree)* | At the moment | Only if low back pain: yes | x | x | x | x | x |
| Pain self-efficacy | At the moment, I can still do things that I enjoy doing, such as hobbies or leisure activity, despite pain.  *1 (I completely disagree) – 2 (I rather disagree) – 3 (I slightly disagree) – 4 (I rather agree) – 5 (I completely agree)* | At the moment | Only if low back pain: yes | x | x | x | x | x |
| **Social support** |  |  |  |  |  |  |  |  |
| Pain-related received support | Since the last questionnaire, have you received support from other people for your low back pain?  *0 (no) – 1 (yes)* | Since the last questionnaire | Only if low back pain: yes | x | x | x | x | x |
| Support provider | Who have you received the support from? *1 (partner) – 2 (family member) – 3 (friend) – 4 (professional caregiver) – 5 (other)* | Since the last questionnaire | Only if low back pain: yes  & Pain-related received support: yes | x | x | x | x | x |
| Received instrumental and emotional support | The person discussed with me possible solutions or did something about the problem. *1 (I completely disagree) – 2 (I rather disagree) – 3 (I slightly disagree) – 4 (I rather agree) – 5 (I completely agree)*  The person comforted or hugged me. *1 (I completely disagree) – 2 (I rather disagree) – 3 (I slightly disagree) – 4 (I rather agree) – 5 (I completely agree)* | Since the last questionnaire | Only if low back pain: yes  & Pain-related received support: yes | x | x | x | x | x |
| **Affect and self-control** |  |  |  |  |  |  |  |  |
| Positive and negative affect | How (…) do you feel at the moment? Excited (Item 1), nervous (Item 2), alert (Item 3), afraid (Item 4), upset (Item 5), inspired (Item 6), distressed (Item 7), annoyed (Item 8), enthusiastic (Item 9), jittery (Item 10), determined (Item 11)  *1 (very slightly or not at all) – 2 (a little) – 3 (moderately) – 4 (quite a bit) – 5 (very much)* | At the moment |  | x | x | x | x | x |
| Self-control | At the moment, I feel like I have no willpower left.  At the moment, I feel balanced.  *1 (fully disagree) –5 (fully agree)* | At the moment |  | x | x | x | x | x |
