# Supplementary material for: Physical activity and psychosocial characteristics of individuals with and without chronic low back pain in daily life: protocol for the PRIA intensive longitudinal study
Source: BMJ Open. 2025 Nov 29;15(11):e109887. doi: 10.1136/bmjopen-2025-109887 (PMC12666212; doi:10.1136/bmjopen-2025-109887)
Supplement: Supplementary file 2 [file bmjopen-2025-109887.reviewer_comments.pdf]

PEER REVIEW HISTORY

BMJ Open publishes all reviews undertaken for accepted manuscripts. Reviewers are asked to complete a checklist review form and are provided with free text boxes to elaborate on their assessment. These free text comments are reproduced below.

ARTICLE DETAILS

Title (Provisional)

Physical activity and psychosocial characteristics of individuals with and without chronic low back pain in daily life: protocol for the PRIA intensive longitudinal study

Authors

Kolodziejczak-Krupp, Karolina; Zipper, Valerie; Wilhelm, Lea O.; Thiel, Lara; Stein, Christoph; Schäfer, Thomas; Pumberger, Matthias; Schmidt, Hendrik; Fleig, Lena

---

VERSION 1 - REVIEW

**Reviewer**	**1**
**Name**	**Dunleavy, Kim**
**Affiliation**	**University of Florida, Physical Therapy**
**Date**	**05-Oct-2025**
**COI**	**None**

The study protocol addresses an extremely important topic with comprehensive ecological movement and self-reported outcomes, novel perspectives, and methods examining individual variability that will provide valuable information for examining chronic back pain from a resource-oriented approach.

Questions for the editor and author:

My only question is related to the timing of the study, which lists the initial enrollment on 01/09/2023 and the end date is 06/09/2025 and whether this fits with BMJ Open's criteria for protocol papers.

There are references are not currently accessible and not sure what the BMJ Open policy is on papers under review:

Reference 16 - Wilhelm et al and Reference 21 Modl et al are currently under review and are not accessible.

Reference 21 - Modl et al - also currently under review

References 60 and 70 may need additional information for book references.

VERSION 1 - AUTHOR RESPONSE

Reviewer: 1
Dr. Kim Dunleavy, University of Florida
Comments to the Author:
The study protocol addresses an extremely important topic with comprehensive ecological movement and self-reported outcomes, novel perspectives, and methods examining individual variability that will provide valuable information for examining chronic back pain from a resource-oriented approach.

>> Response: We very much appreciate the positive evaluation of our protocol.

Questions for the editor and author:
My only question is related to the timing of the study, which lists the initial enrollment on 01/09/2023 and the end date is 06/09/2025 and whether this fits with BMJ Open's criteria for protocol papers. [NOTE FROM THE EDITORS: As the protocol manuscript was submitted to us before the study completion, we remain able to consider, so long as no results have been reported. For future reference, however, please note that we prefer submission of protocols at as early as stage as possible. Please ensure the study status and the expected timeline for completion/reporting of the results is clear in the manuscript]

>> Response: We agree that the originally specified study end date could raise questions regarding study timing. As recruitment and data analyses are still ongoing, we have updated the planned study end date to 01/06/2026 in both the protocol (see p. 20) and the German Clinical Trials Register (DRKS00032978).

For future projects, we will certainly keep in mind to submit our protocol at an earlier stage in order to align more closely with the journal's policy regarding the timing of protocol submissions.

There are references are not currently accessible and not sure what the BMJ Open policy is on papers under review:
Reference 16 - Wilhelm et al and Reference 21 Modl et al are currently under review and are not accessible.
Reference 21 - Modl et al - also currently under review
References 60 and 70 may need additional information for book references.

>> Response: Thank you for your careful review. We have clarified the status of the manuscripts '16' and '21' accordingly. The paper previously cited as reference '16' (Wilhelm et al., 2025) has since been published, and we have updated the reference to reflect this. The manuscript previously listed as reference '21' (Mödl et al., 2025) is still under review; therefore, we have removed this reference from the manuscript.

Regarding the remaining references, please note that reference '60' (Fydrich et al., 2007) refers to a published questionnaire rather than a book or book chapter; therefore, it follows a different citation format. For reference '70' (Cutrona & Russell, 1990), we have now added the missing citation details.

>> Please note that we have implemented additional changes requested by the journal after submitting the revised manuscript. Specifically, we have provided an overview of the EMA sampling schedule and EMA measures in Table S1 of the Online Supplement (previously Table 2 in the main document). Consequently, we now refer to Table S1 (previously Table 2) in the main document.
